# Supplementary material for: A third-generation mouse model of Alzheimer's disease shows early and increased cored plaque pathology composed of wild-type human amyloid β peptide
Source: J Biol Chem. 2021 Jul 27;297(3):101004. doi: 10.1016/j.jbc.2021.101004 (PMC8397900; doi:10.1016/j.jbc.2021.101004)
Supplement: Figures S1–S3 [file mmc1.pdf]

Supplementary Figures

A 3rd generation mouse model of Alzheimer's disease shows early and increased cored plaque pathology composed of wild-type human amyloid β peptide

Kaori Sato[1,2], Naoto Watamura[1], Ryo Fujioka[1], Naomi Mihira[1], Misaki Sekiguchi[1], Kenichi Nagata[3], Toshio Ohshima[2], Takashi Saito[4], Takaomi C. Saido[1,*] and Hiroki Sasaguri[1,*]

[1]Laboratory for Proteolytic Neuroscience, RIKEN Center for Brain Science, 2-1 Hirosawa, Wako, Saitama 351-0198, Japan

[2]Laboratory for Molecular Brain Science, Department of Life Science and Medical Bioscience, Waseda University, Shinjuku, Tokyo 162-8480, Japan

[3]Department of Functional Anatomy and Neuroscience, Nagoya University Graduate School of Medicine, Nagoya, Aichi 466-8550, Japan

[4]Department of Neurocognitive Science, Institute of Brain Science, Nagoya City University Graduate School of Medical Sciences, Nagoya, Aichi 467-8601, Japan

Supplementary Figure 1.

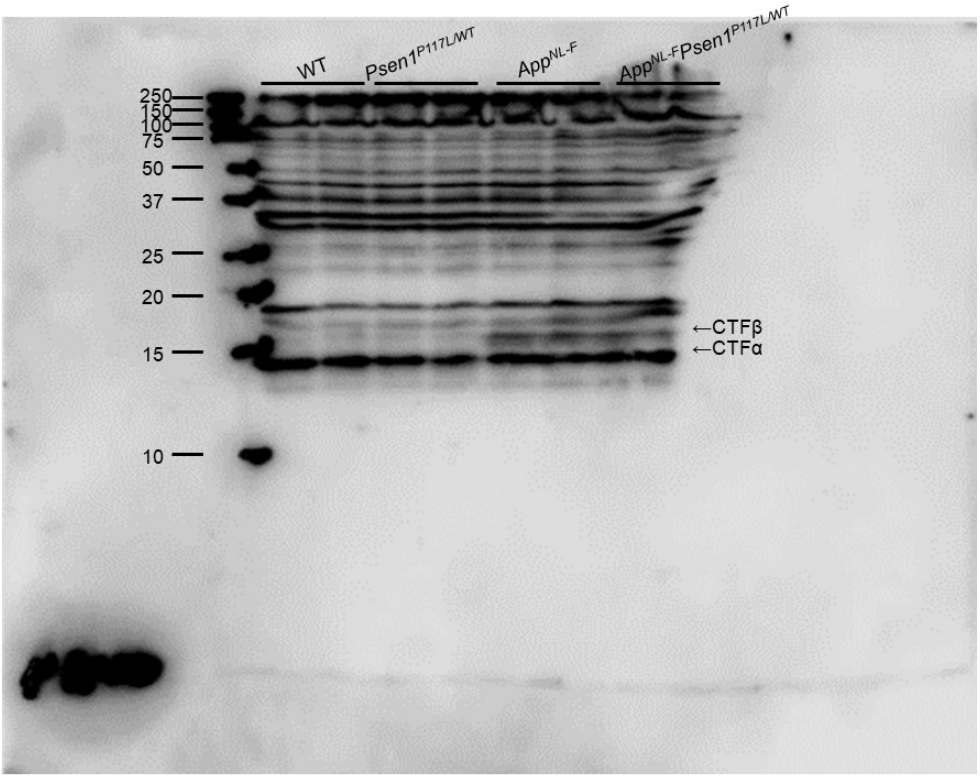

Full Western blots of CTFs shown in Figure 1a.

Supplementary Figure 2.

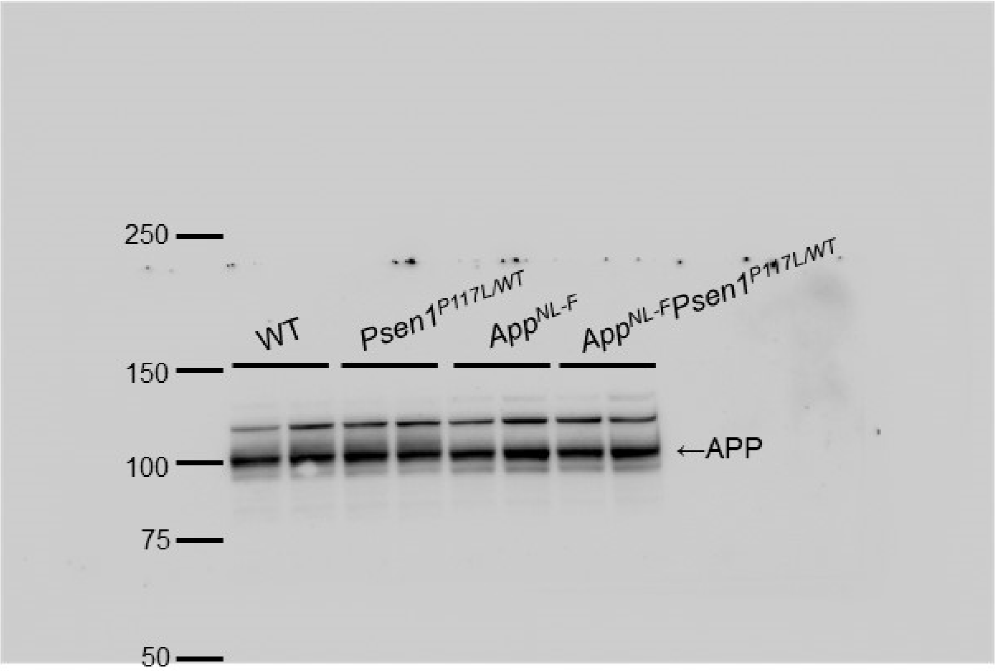

Full Western blots of APP shown in Figure 1a.

Supplementary Figure 3.

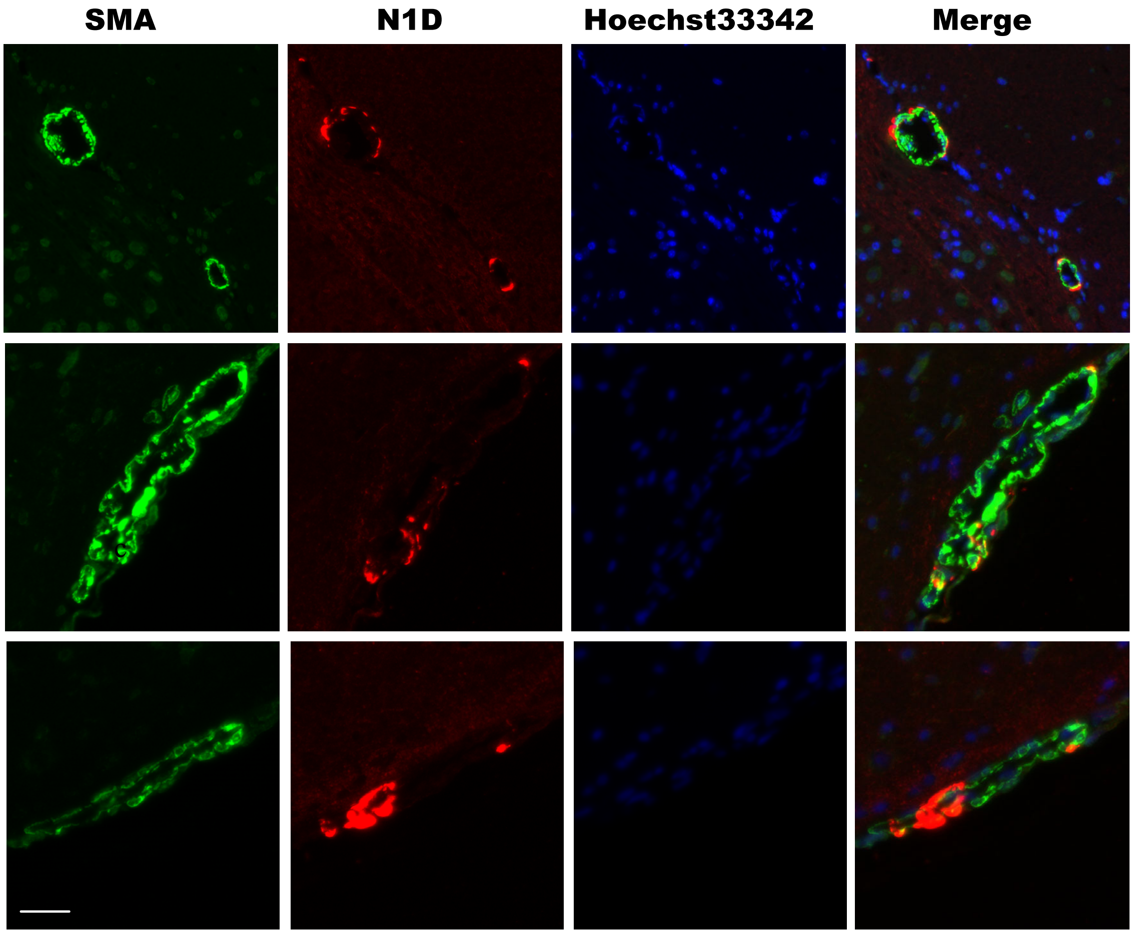

Representative images of CAA in the meningeal blood vessels of 12-month-old App^{NL-F}×$Psen1^{P117L}$ mice. SMA (smooth muscle actin) marks blood vessels (green). N1D represents Aβ deposition (red). Hoechst33342 binds to dsDNA (blue). The Scale bar indicates 25 μm.
